# Wild Barley Exhibits Higher Phosphorus‐Use Efficiency and Greater Rhizosheath Carboxylates Than Cultivated Barley Under Low‐Phosphorus Conditions

**DOI:** 10.1111/ppl.70990

**Published:** 2026-06-30

**Authors:** Yunpeng Tao, Xiaowen Fan, Tahmina Nazish, Jiayin Pang, Meixue Zhou, Fanrong Zeng, Zhong‐Hua Chen, Sergey Shabala

**Affiliations:** ^1^ School of Biological Sciences, the University of Western Australia Perth Australia; ^2^ The UWA Institute of Agriculture, the University of Western Australia Perth Australia; ^3^ Tasmania Institute of Agriculture, University of Tasmania Hobart Australia; ^4^ MARA Key Laboratory of Sustainable Crop Production in the Middle Reaches of the Yangtze River, College of Agriculture, Yangtze University Jingzhou China; ^5^ School of Agriculture, Food and Wine, Waite Research Institute, Adelaide University Glen Osmond Australia; ^6^ International Research Centre for Environmental Membrane Biology, Foshan University Foshan China

## Abstract

Phosphorus (P) deficiency constrains cereal production, yet improving low‐P performance remains challenging because P efficiency depends on rhizosphere mobilisation and internal utilisation. Wild relatives may provide insights into traits weakened during domestication. Here, we compared cultivated barley ( *Hordeum vulgare* ; CB) and wild barley ( *H. spontaneum* ; WB) to identify traits underpinning low‐P tolerance. Twenty barley genotypes (10 CB and 10 WB) were grown under low (P5) and moderate (P20) P supply, and twelve low‐P response physiological traits were integrated into a composite tolerance index (D‐value) to assess the multidimensional basis of low‐P tolerance. Under both P5 and P20, WB showed consistently higher physiological P‐use efficiency (PPUE) than CB, while root morphological traits were broadly similar between CB and WB. This decoupling of biomass production from tissue P status indicates more efficient internal utilisation of absorbed P in WB. Under P5, the amount of rhizosheath citrate per plant was strongly stimulated and showed a pronounced species effect with WB exhibiting significantly higher rhizosheath citrate than CB. By contrast, increasing P supply shifted carboxylate composition towards malate dominance (85% at P20). The D value showed strong and consistent associations with key growth, PPUE, root morphology and rhizosphere functional traits in both CB and WB, validating its effectiveness as an integrative metric of low‐P tolerance. Overall, WB's superior low‐P performance is primarily driven by higher PPUE and elevated citrate exudation under acute P limitation, highlighting the value of targeting internal P utilisation and rhizosphere mobilisation efficiency to improve P‐use efficiency in barley.

## 1 Introduction

Phosphorus (P) deficiency remains a major constraint on cereal production because most soil P is present in biologically unavailable forms, while agriculture simultaneously depends on finite phosphate rock inputs and faces growing pressure to improve P‐use efficiency (PUE) to reduce costs and environmental losses (MacDonald et al. 2011). In the rhizosphere, plants can enhance P availability by exuding carboxylates that modify local soil chemistry. These organic anions can mobilise phosphate bound to Fe and Al oxides in acidic soils through chelation and ligand exchange, while in alkaline soils they can increase the solubility of Ca‐associated phosphates by complexing Ca^2+^ and reducing Ca activity in solution (Bolan et al. 2025). Overall, PUE consists of two key components: P‐acquisition efficiency, that is, the ability to take up P from soil, and internal P‐utilisation efficiency, namely how efficiently plants convert a given P investment into biomass, which can be captured by indices such as physiological P‐use efficiency (PPUE) (Han et al. 2022; Hazarika, Dehmer, et al. 2025).

Despite extensive work on P‐acquisition traits (root morphology, exudation, phosphatases) and internal P‐utilisation traits (PPUE, P remobilisation), linking these dimensions into a coherent mechanistic framework that explains genotypic performance remains a key challenge for breeding P‐efficient crops (van de Wiel et al. 2016). One key reason is the strong context dependence of many so‐called “P‐efficiency traits”. For example, enhanced root length or surface area does not consistently translate into greater biomass under low‐P conditions when soil P is tightly sorbed and diffusion‐limited, as reported for barley and wheat genotypes grown in low‐P sandy soils (Heuer et al. 2017; Rose et al. 2013). Similarly, high carboxylate exudation does not always improve plant growth if exudation is not coordinated with internal P‐utilisation capacity or if mobilised P cannot be efficiently taken up and retained by the plant (Pearse et al. 2007; Wang et al. 2025). Recent studies in cereals further show that genotypes with comparable P uptake can differ markedly in biomass production because of differences in PPUE and P allocation within tissues, indicating that acquisition traits alone are insufficient predictors of low‐P performance (Ampong et al. 2025; Sillo et al. 2025). Consequently, integrative approaches that simultaneously consider growth, root morphology, rhizosphere processes and internal P‐utilisation traits are increasingly recognised as essential for identifying robust low‐P tolerance syndromes, rather than relying on single indicators in isolation.

Crop domestication adds another layer of complexity. Selection under high‐input, fertilised agroecosystems tends to prioritise rapid growth and responsiveness to nutrient supply, which can relax selection for traits conferring efficiency and resilience under chronic nutrient scarcity (Nimmo et al. 2023). This raises a practical and timely question for barley improvement: do wild relatives retain belowground useful strategies, particularly rhizosphere P mobilisation and efficient internal P‐utilisation, that can be leveraged to enhance performance under low‐input or P‐deficient conditions? Previous studies have shown that Tibetan wild barley ( *Hordeum spontaneum* ) differs from cultivated barley ( *Hordeum vulgare* ) in its physiological responses to P limitation, including lower tissue P concentrations, greater maintenance of growth under low P, and altered root–shoot allocation patterns (Nadira et al. 2014; Cai et al. 2022). Moreover, Tibetan wild barley exhibits enhanced expression of genes associated with P starvation signalling and internal Pi recycling, including those regulating P remobilisation from older tissues, coupled with high plasticity in root functional traits and root–shoot P allocation, indicating that low‐P tolerance is primarily supported by an efficient internal P economy rather than elevated P acquisition capacity (Long et al. 2019). Yet, direct, side‐by‐side tests that integrate rhizosheath carboxylates, phosphatase activity, root morphology, and internal P‐utilisation efficiency within the same experimental framework remain limited, especially when paired with an objective multi‐trait ranking of low‐P tolerance.

Here, we addressed these knowledge gaps by evaluating 20 barley genotypes (10 cultivated, 10 wild) selected from a broader screening panel for contrasting low‐P performance (Tao et al. 2026). Plants were grown under two P supplies (P5 and P20) to determine phenotypes such as biomass allocation, root morphology, shoot P concentration, PPUE, and root physiological parameters (exudation of carboxylates and acid phosphatase). We then integrated twelve low‐P response indicators into a composite tolerance index (D value) to rank genotypes within cultivated (CB) and wild barley (WB), and to test whether multi‐trait integration yields a more consistent representation of low‐P tolerance than any single trait. Specifically, we asked: (1) whether WB can release more carboxylates and higher PPUE under P deficiency; (2) whether the proportion of citrate (tricarboxylates) decreases with increasing P supply, indicating a shift in carboxylate composition to maintain carbon balance; and (3) whether the composite D value captures a coherent low‐P tolerance syndrome supported by coordinated correlations among growth, P‐use and rhizosphere traits.

## 2 Materials and Methods

### 2.1 Plant Materials and Growth Conditions

A subset of 20 barley genotypes exhibiting contrasting grain yield under low‐P conditions was selected from 96 accessions evaluated in a previous experiment (Tao et al. 2026). This subset comprised 10 CB and another 10 WB genotypes, representing both low‐P tolerant and low‐P sensitive phenotypes within each species.

The experiment was conducted in an environment‐controlled greenhouse at the University of Western Australia, Perth, Australia, with mean day/night temperatures of approximately 21/16°C and an average relative humidity of 62%. Plants were grown individually in 1 L black plastic pots containing 1.1 kg of a low‐nutrient growth medium consisting of field soil collected from Cunderdin Agriculture College (31.64°S, 117.24°E) mixed with washed river sand at a 1:9 (w/w) ratio (soil volume: 0.9 L per pot). Soil chemical properties were analyzed by CSBP FutureFarm Analytical Laboratories (Bibra Lake, Australia) and included 1 μg g^−1^ NH_4_^+^–N, 1 μg g^−1^ NO_3_^−^–N, 4 μg g^−1^ Colwell‐P, 47 μg g^−1^ Colwell‐K, 0.1% organic carbon, pH (CaCl_2_) 6.4, and a phosphorus buffering index of 7.9.

Two P treatments were applied: low phosphorus (P5; 5 μg P g^−1^ soil) and moderate phosphorus (P20; 20 μg P g^−1^ soil), supplied as KH_2_PO_4_. Prior to sowing, all pots received a basal nutrient solution containing: 33.75 μg N g^−1^ soil as Ca(NO_3_)_2_·4H_2_O; 11.25 μg N g^−1^ soil as NH_4_Cl; 45 μg S g^−1^ soil as K_2_SO_4_; 2 μg Zn g^−1^ soil as ZnSO_4_·7H_2_O; 4 μg Mn g^−1^ soil as MnSO_4_·H_2_O; 0.5 μg Cu g^−1^ soil as CuSO_4_·5H_2_O; 0.4 μg Mo g^−1^ soil as Na_2_MoO_4_·2H_2_O; and 3 μg Fe g^−1^ soil as FeNaEDTA.

Seeds were sown in early May 2025, with three seeds per pot, and seedlings were thinned to one healthy plant per pot 12 days after sowing. The soil mixture was initially adjusted to 80% of pot capacity (PC), with 100% PC corresponding to a gravimetric water content of 12% (w/w), determined prior to the experiment following Pang et al. (2017). Throughout the experiment, soil moisture was maintained at 80% PC by weighing pots and replenishing water with deionized water every 2 days. Plants were harvested after 40 days of growth. Tiller number and leaf SPAD values were recorded 1 day before harvest. SPAD measurements were taken on the flag leaf at approximately one‐third of the distance from the leaf tip.

The experiment followed a two‐factorial design with two phosphorus levels and two species, with genotype nested within species. Each genotype × *p* treatment combination included four biological replicates, and pots were arranged in a completely randomized design. Genotype was treated as a random factor nested within species in all subsequent statistical analyses.

### 2.2 Root Exudates Collection and Quantification

Barley plants were harvested 40 days after sowing for the collection of rhizosheath‐derived carboxylates. At harvest, plants were carefully removed from the pots, and loosely attached sand–soil material was gently shaken off. Soil that remained firmly attached to the root surface was defined as rhizosheath soil. The intact root system, together with the adhering rhizosheath, was transferred into a beaker containing a known volume of 0.2 mM CaCl_2_ solution to preserve cellular integrity. The suspension was gently stirred to release the rhizosheath soil into solution. This rhizosheath extraction procedure was adapted from established protocols (Pang et al. 2022). After extraction, roots were removed, and the resulting rhizosheath suspension was divided for subsequent analyses. Two 0.5 mL aliquots were transferred into 2 mL centrifuge tubes for the determination of acid phosphatase activity (Wen et al. 2019). For carboxylate quantification, approximately 1.5 mL of the rhizosheath suspension was passed through a 0.45 μm syringe filter into 2 mL High Performance Liquid Chromatography (HPLC) vials, acidified with a small volume of orthophosphoric acid (H_3_PO_4_) to minimize microbial activity. Filtered samples were stored at −20°C prior to analysis. Carboxylate concentrations were determined using high‐performance liquid chromatography according to standard procedures (Cawthray 2003).

### 2.3 Root Morphology Analysis

After root exudate collection, entire root systems were thoroughly washed with deionized water to remove adhering soil particles. Roots were carefully spread in a transparent tray containing a thin layer of water to minimize overlap and scanned in their entirety using an Epson Expression 1680 flatbed scanner at a resolution of 400 dpi (Epson America). Scanned root images were analysed using WinRHIZO Pro software (Regent Instruments Inc.) to quantify root morphological traits, including total root length (TRL), root surface area (RSA), and mean root diameter (MRD). Specific root length (SRL) was calculated as the ratio of total root length to root dry weight (Pang et al. 2022).

### 2.4 Shoot P Concentration Determination

Oven‐dried shoot samples were finely ground using a Geno/Grinder 2000 (Spex SamplePrep, Metuchen). Approximately 100 mg of ground plant material was digested using a concentrated nitric acid–perchloric acid mixture (3:1, v/v) under heating until complete digestion. Following digestion, P concentration in shoot tissues was determined colorimetrically using the malachite green method (Pang et al. 2025). Shoot phosphorus concentration (shoot [P]) was expressed on a dry weight basis. Shoot P content was calculated by multiplying shoot [P] by shoot dry weight for each individual plant. Physiological phosphorus‐use efficiency (PPUE) was expressed as the ratio of shoot dry weight to shoot [P], following the approach described by Pang et al. (2018).

### 2.5 Construction of the Composite Low‐P Tolerance Index (D‐Value)

To integrate multiple traits associated with P deficiency responses, a composite low‐P tolerance index (D value) was constructed following a weighted membership function approach described previously (Zou et al. 2020), with modifications to accommodate low‐P responses in barley. Twelve indicators reflecting plant growth, P acquisition and utilization, root morphology and physiological traits were included in the calculation: PPUE, shoot dry weight (SDW), root dry weight (RDW), shoot P concentration (Shoot [P]), tiller number (TN), SPAD value, total root length (TRL), root surface area (RSA), mean root diameter (MRD), specific root length (SRL), acid phosphatase activity (APase), and total carboxylate exudation per plant (Carb). For traits negatively associated with low‐P tolerance (e.g., Shoot [P] and MRD), values were direction‐adjusted prior to analysis so that higher values consistently indicate greater tolerance. All trait values were first normalized using a min–max transformation to generate dimensionless membership values ranging from 0 to 1. Trait weights were then determined based on the relative variability of each indicator across genotypes, calculated from their coefficients of variation. The composite D value for each genotype was obtained as the weighted sum of the corresponding membership values across all traits. Detailed equations for membership function normalisation, weight determination and D‐value calculation are provided in Wang et al. (2023). Higher D values indicate overall greater low‐P tolerance, representing coordinated advantages in biomass accumulation, P‐utilization efficiency, root morphological and physiological traits under low‐P conditions.

### 2.6 Statistics

All statistical analyses were performed using R software (version 4.4.2; R Core Team). For each parameter, effects of P level and species (CB; WB) were analyzed using a two‐factorial nested linear mixed‐effects model, in which *P level* and *species* were treated as fixed effects, and *genotype* was treated as a random effect nested within species. Significance of fixed effects was assessed using analysis of variance (ANOVA) based on the mixed‐effects model. Main effects of P level (P), species (S), and their interaction (*P* × S) are reported in the figures using standard significance codes (*p* < 0.05; *p* < 0.01; *p* < 0.001; ns, not significant). Pearson correlation analyses were conducted to examine relationships among biomass traits, P acquisition and utilization, root morphological and physiological traits under low‐P conditions. Correlation matrices were visualized using correlograms, with correlation coefficients and significance levels indicated.

## 3 Results

### 3.1 Biomass Allocation and Growth Responses to P Supply in Cultivated and Wild Barley

**FIGURE 1:**
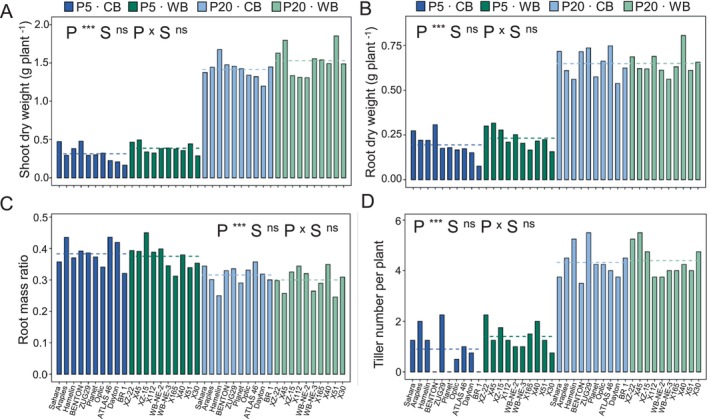
(A) Shoot dry weight, (B) root dry weight, (C) root mass ratio, and (D) tiller number per plant in 10 domesticated barley (CB) accessions and another 10 wild barley (WB) accessions grown under low phosphorus (P5) and moderate phosphorus (P20) conditions for 40 days. Bars represent individual genotype, with dashed horizontal lines indicating the mean value for each species × phosphorus level combination. Statistical significance of the main effects of phosphorus level (P), species (S), and their interaction (*P* × S) was tested using a two‐factorial nested analysis of variance, with genotype nested within species as a random effect. Significance levels are indicated as: ***p < 0.001; *p < 0.05; ns, not significant.

Phosphorus supply had a strong and consistent effect on barley growth across all measured traits (Figure 1). Compared with the low‐P treatment (P5), moderate P supply (P20) significantly increased shoot dry weight (SDW) and root dry weight (RDW) in both CB and WB genotypes (Figure 1A,B; *p* < 0.001). Tiller number per plant and SPAD values were also markedly higher under P20 than under P5, indicating enhanced vegetative growth and leaf chlorophyll status with improved P availability (Figure 1D; Figure S1). In contrast, species effects were generally weak. No significant differences between CB and WB were detected for SDW, RDW, root mass ratio (RMR) or tiller number (TN) when averaged across P levels (Figure 1A–D). Root mass ratio decreased under P20 relative to P5 in both CB and WB, reflecting a shift in biomass allocation from belowground to aboveground organs with increasing P supply (Figure 1C; *p* < 0.001). A significant interaction between P level and species was observed only for SPAD values (Figure S1; *p* < 0.05), suggesting a differential chlorophyll response of CB and WB to P availability. Despite the absence of strong species effects, substantial genotypic variation was evident within both CB and WB for all growth‐related traits under both P levels.

### 3.2 Divergent Root Morphological Strategies Under Contrasting P Availability

**FIGURE 2:**
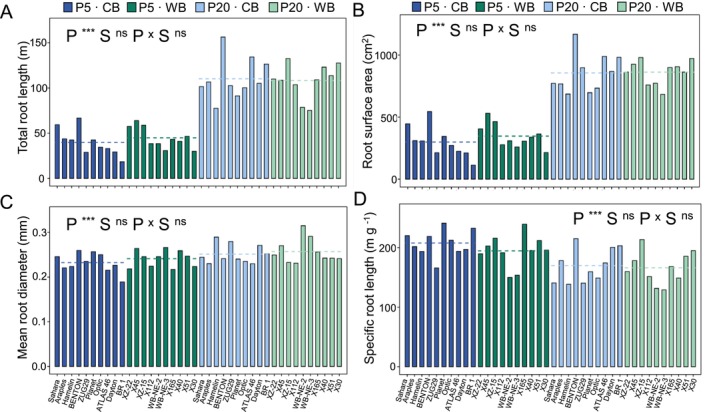
Root morphological traits, including (A) total root length, (B) root surface area, (C) mean root diameter, and (D) specific root length in 10 domesticated barley (CB) accessions and another 10 wild barley (WB) accessions grown under low phosphorus (P5) and moderate phosphorus (P20) conditions for 40 days. Bars represent individual genotypes, and dashed horizontal lines indicate the mean value for each species × phosphorus level combination. The significance of the main effects of phosphorus level (P), species (S), and their interaction (*P* × S) was assessed using a two‐factorial nested analysis of variance, with genotype nested within species as a random effect. Significance levels are indicated as: ***p < 0.001; ns, not significant.

Root morphological traits responded strongly to P availability across both CB and WB genotypes (Figure 2). P20 significantly increased TRL and RSA compared with the P5 in both CB and WB (Figure 2A,B; *p* < 0.001). In contrast, SRL was significantly reduced under P20 relative to P5 (Figure 2D; *p* < 0.001). Mean root diameter exhibited a slight increase under P20 compared with P5 (Figure 2C; *p* < 0.001). However, no significant species effect was detected for all root morphological traits, including TRL, RSA, MRD, or SRL (Figure 2A–D). Likewise, no significant interaction between P level and species was observed for these traits (Figure 2), indicating that CB and WB exhibited similar root morphological plasticity in response to changes in P supply. Despite the lack of significant differences between CB and WB, substantial genotypic variation was evident within both CB and WB groups under both P levels, particularly for TRL and SRL (Figure 2A,D).

### 3.3 Phosphorus Uptake and Physiological P‐Use Efficiency in Response to P Supply

**FIGURE 3:**
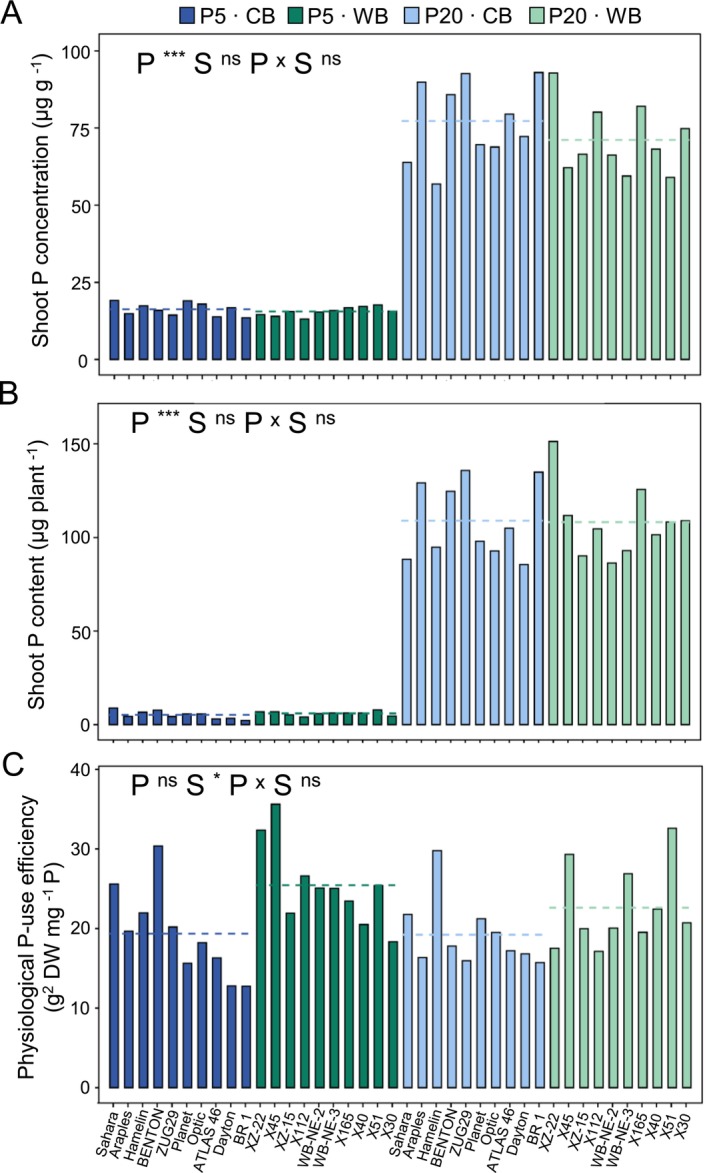
(A) Shoot phosphorus concentration, (B) shoot phosphorus content per plant, and (C) physiological phosphorus‐use efficiency in 10 domesticated barley (CB) accessions and another 10 wild barley (WB) accessions grown under low phosphorus (P5) and moderate phosphorus (P20) conditions for 40 days. Bars represent individual genotype, with dashed horizontal lines indicating the mean value for each species × phosphorus level combination. The significance of the main effects of phosphorus level (P), species (S), and their interaction (*P* × S) was tested using a two‐factorial nested analysis of variance, with genotype nested within species as a random effect. Significance levels are indicated as follows: ***p < 0.001; *p < 0.05; ns, not significant.

Both shoot P concentration and content were strongly affected by P supply, whereas species effects and *P* × species interactions were not significant (Figure 3A,B). Across both CB and WB, plants grown under P20 exhibited markedly higher shoot P concentration and shoot P content compared with those grown under P5 (*p* < 0.001). In contrast, mean shoot P concentration and shoot P content did not differ significantly between CB and WB at either P level. Physiological P‐use efficiency showed a contrasting response pattern (Figure 3C). While PPUE was not significantly influenced by P supply, a significant species effect was observed (*p* < 0.05), with WB consistently exhibiting higher PPUE than CB under both P levels. Together, these results indicate that WB achieves greater biomass production per unit of shoot P concentration.

### 3.4 Rhizosheath Carboxylates and Activity of Acid Phosphatases

**FIGURE 4:**
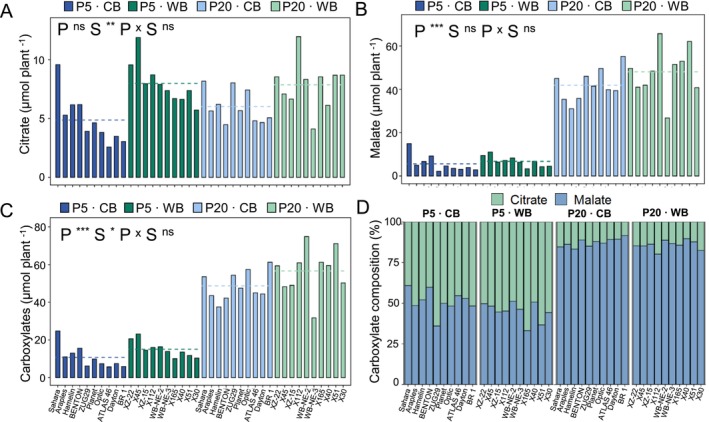
The amount of (A) citrate, (B) malate and (C) total carboxylates recovered from rhizosheath soil per plant, and (D) carboxylate composition expressed as the percentage of citrate and malate in 10 domesticated barley (CB) accessions and another 10 wild barley (WB) accessions grown under low phosphorus (P5) and moderate phosphorus (P20) conditions for 40 days. Bars represent individual genotype, and dashed horizontal lines indicate the mean value for each species × phosphorus level combination. The significance of the main effects of phosphorus level (P), species (S), and their interaction (*P* × S) was assessed using a two‐factorial nested analysis of variance, with genotype nested within species as a random effect. Significance levels are indicated as: ***p < 0.001; **p < 0.01; *p < 0.05; ns, not significant.

The amount of carboxylates recovered from rhizosheath soil per plant responded differently to P supply (Figure 4). The amount of citrate recovered in rhizosheath per plant showed significant effects from species (*p* < 0.01) (Figure 4A). Overall, the amount of citrate in rhizosheath of WB was substantially greater than CB, and the difference between CB and WB was more pronounced under P5 than under P20, with WB releasing 39% more citrate than CB at P5, whereas citrate release in WB was only 29% higher than CB under P20. In contrast, the amount of malate in rhizosheath per plant was strongly stimulated by increased P level, being almost doubled at P20 than P5 (Figure 4B; *p* < 0.001). The total amount of carboxylates per plant increased significantly at P20 relative to P5 (Figure 4C; *p* < 0.001) and also differed between CB and WB (Figure 4C; *p* < 0.05), indicating that there are differences in the overall amount of rhizosheath carboxylates between CB and WB, which are not affected by the *P* × species interaction. Carboxylate composition further revealed a clear shift in dominant anions between P levels (Figure 4D). Under P5, citrate and malate contributed about equal proportions to the total carboxylates in both CB and WB. Under P20, however, the profile shifted toward malate dominance, with malate accounting for about 85% of total carboxylates and citrate representing only about 15%. Acid phosphatase activity was strongly affected by P supply (Figure S2; *p* < 0.001), with markedly higher activities under P5 than under P20 in both CB and WB. While species effects were not statistically significant, WB tended to maintain higher acid phosphatase activity under P5 across most genotypes.

### 3.5 Integrated Trait Networks and Genotypic Ranking of Low‐P Tolerance

#### 3.5.1 Genotypic Ranking of Low‐P Tolerance Using a Composite Index

**FIGURE 5:**
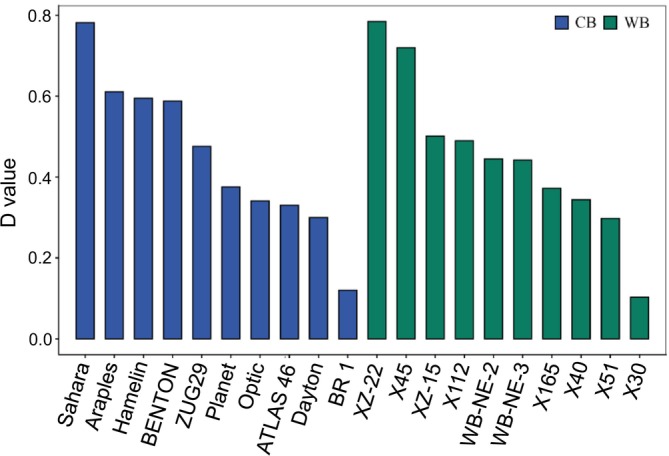
Integrated ranking of low‐phosphorus tolerance for 10 cultivated barley (CB) and another 10 wild barley (WB) genotypes based on a composite tolerance index (D value). The D value was calculated by integrating multiple low‐phosphorus–responsive traits, including growth, root morphological traits, phosphorus‐acquisition and ‐utilisation efficiency, and root physiological traits. Bars represent individual genotypes within each species, ranked in descending order of D value, with higher values indicating greater tolerance to low‐phosphorus availability.

To visualise genotypic variation in low‐P tolerance, a composite tolerance index (D value) was calculated separately for both CB and WB, and genotypes were ranked within CB and WB (Figure 5). Within CB, the D value varied substantially across genotypes, allowing a clear separation of relatively tolerant and sensitive cultivated accessions under P deficiency. “Sahara” showed the highest D‐value among CB genotypes, followed by “Arables,” “Hamelin,” and “BENTON,” whereas “BR1” ranked lowest, indicating strong low‐P sensitivity within the cultivated panel. Within WB, a similar range of D values was observed, highlighting pronounced genotypic diversity in low‐P tolerance among wild accessions. “XZ‐22” and “X45” ranked highest within WB, while “X30” displayed the lowest D‐value, representing the most sensitive accession.

#### 3.5.2 Trait Networks Underlying Low‐P Tolerance Differ Between CB and WB

**FIGURE 6:**
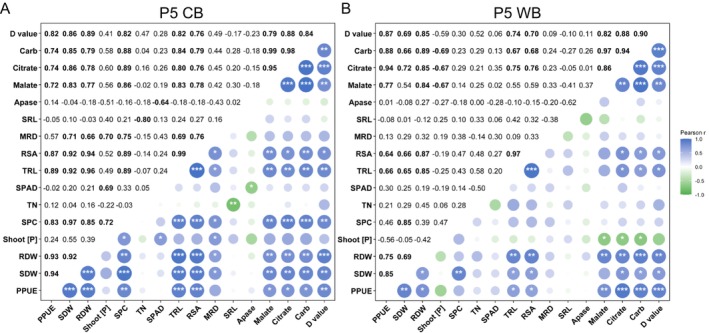
Pairwise Pearson correlations among growth, root morphological, physiological traits and physiological phosphorus‐use efficiency under low‐phosphorus (P5) conditions in cultivated barley (CB; A) and wild barley (WB; B). The lower triangle shows circles with significant marks, while the upper triangle shows the correlation coefficients. Circle size represents the absolute value of the Pearson correlation coefficient (|r|), while color indicates the direction and magnitude of the correlation (blue, positive; green, negative). Asterisks denote statistically significant correlations (*p < 0.05; **p < 0.01; ***p < 0.001). APase, acid phosphatase activity normalised to root dry weight; Carb, total carboxylates per plant recovered in the rhizosheath soil; Citrate, citrate exudation per plant; D value, composite low‐phosphorus tolerance index; Malate, malate exudation per plant; MRD, mean root diameter; PPUE, physiological phosphorus‐use efficiency; RDW, root dry weight; RSA, root surface area; SDW, shoot dry weight; Shoot [P], shoot phosphorus concentration; SPAD, chlorophyll index; SPC, shoot phosphorus content; SRL, specific root length; TN, tiller number; TRL, total root length.

Correlation analyses under P5 showed that the D‐value was consistently and positively associated with a suite of key low‐P responsive traits in both CB and WB (Figure 6). In both CB and WB, the D‐value exhibited significant positive correlations with PPUE, SDW, RDW, and major root morphological traits, including TRL and RSA. In addition, the D‐value was strongly linked to root physiological traits involved in P mobilisation, particularly the amount of malate, citrate, and total carboxylates per plant. These relationships were broadly comparable between CB and WB, indicating that the composite index captured common trait dimensions underpinning low‐P tolerance in both CB and WB. Importantly, the simultaneous associations of the D‐value with biomass production, physiological P‐use efficiency, root system development, and root exudation traits demonstrate that this index integrates multiple physiological and morphological strategies related to low‐P adaptation. The strong and coherent correlation structure reflects the integrated nature of the D‐value, noting that correlations between biomass and root morphological traits are partly expected given that these variables contribute directly to the index. Some correlations between traits did not directly drive the index, such as PPUE and root exudation traits, providing more independent support for its use as a metric for genotypic ranking in low‐P tolerance.

## 4 Discussion

### 4.1 Higher Physiological P‐Use Efficiency in Wild Barley Reflects a Distinct Internal P Utilisation

Under both P5 and P20, WB consistently maintained higher PPUE than CB, despite broadly comparable shoot P concentrations and major root morphological traits (Figures 2, 3). This combination, greater biomass per unit tissue P without an accompanying increase in tissue P status or morphological foraging capacity, points to a WB advantage that is more strongly attributable to internal P utilization than to enhanced P acquisition. A similar pattern has been reported beyond cereals: in a screening of cultivated and wild potato germplasm, several wild *Solanum* accessions maintained biomass under low P through efficient internal P use, and one accession achieved high biomass despite lower P uptake (Hazarika, Ashfaq, et al. 2025). In cereals, this “same tissue P, different growth” decoupling is well recognized, with genotypic differences in yield or biomass under P limitation often better explained by P utilization efficiency than by uptake alone (Manske et al. 2001; McDonald et al. 2015). Mechanistically, we speculate that higher PPUE at comparable shoot P concentration may reflect one or more downstream processes that reduce the effective P cost of growth, including tighter partitioning of cellular P away from low‐return pools, more efficient remobilization and recycling of Pi, and metabolic and membrane‐lipid reprogramming such as phospholipid replacement and the use of PPi‐dependent bypasses (Veneklaas et al. 2012; Heuer et al. 2017; Han et al. 2022; Lambers 2022). These mechanisms are conceptually aligned with evidence from Tibetan wild barley, where enhanced P‐starvation signaling and internal Pi recycling were associated with improved low‐P performance without requiring a larger root foraging footprint (Long et al. 2019). Collectively, our results are consistent with the view that WB achieves low‐P resilience through a distinct internal P utilization strategy, one that preserves growth at similar tissue P status. These findings point to internal P allocation and recycling as possible processes worthy of further investigation in the context of exploiting WB germplasm to improve P‐use efficiency.

### 4.2 Enhanced the Amount of Rhizosheath Citrate as a Rhizosphere P‐Mobilisation Advantage in Wild Barley

The amount of rhizosheath carboxylates per plant showed a clear P‐dependent reallocation in both magnitude and composition (Figure 4). The amount of citrate in rhizosheath per plant increased strongly under P5 and displayed significant species effects and a *P* × species interaction; under P5, WB exhibited 39% more citrate than CB (with a smaller difference at P20), indicating that the WB citrate advantage is most pronounced under acute P limitation. A similar P‐dependent suppression of citrate release with increasing P supply has been reported in other species; for example, in *Macadamia integrifolia* , citrate exudation was highest under 0 and 5 μM P and progressively declined as external P levels increased (Pan et al. 2025). By contrast, the amount of rhizosheath malate per plant mainly increased with P supply (nearly doubling at P20) and showed no species effect, such that total carboxylates were higher at P20 than P5, supporting the point that carboxylate release does not necessarily scale monotonically with the severity of P deficiency, but instead reflects a rebalancing among constraints and functions, including carbon costs and strong rhizosphere turnover/sorption processes. This non‐monotonic response is not unique: in *Kennedia nigricans* , total carboxylate exudation was highest at moderate P supply and declined at both lower and higher P levels, although this pattern was species‐dependent, as the other two species in the same study did not show the same trend (Suriyagoda et al. 2012). The compositional shift reinforces this interpretation: citrate and malate contributed broadly similar proportions under P5, whereas malate dominated under P20 (85%) and citrate declined to 15% (Figure 4D). This switch is consistent with established frameworks in which plants adjust the “type” of low‐molecular‐weight organic anions released depending on external P availability and the trade‐off between P‐mobilization effectiveness and the cost/constraints of sustained exudation. A similar pattern of carboxylate composition responding to P availability is observed in wheat under field conditions. In a field study assessing rhizosphere organic anions across different growth stages, wheat grown under P0 (no P fertilization) exhibited a higher proportion of citrate relative to dicarboxylic acids such as malate and succinate, whereas under P48 (48 kg P ha^−1^) conditions, the proportion of dicarboxylic acids increased substantially (Wang et al. 2017). Mechanistically, citrate (a tricarboxylate) is often a more effective P‐mobilizing ligand than dicarboxylates such as malate because its higher anionic charge density and strong complexation with Fe/Al can promote ligand exchange and ligand‐enhanced dissolution on metal (hydr)oxide surfaces, thereby releasing sorbed phosphate; comparative syntheses explicitly identify citrate as among the most powerful P‐desorbing organic acids, whereas malate is generally less potent (Andrino et al. 2021; Santoro et al. 2024). Taken together, our data suggest that under P5, WB likely enhance rhizosphere P‐mobilization potential primarily through an elevated amount of rhizosheath citrate, whereas under P20 the system shifts towards a malate‐dominated profile that may be more compatible with maintaining carbon metabolic and charge balance when low P stress is alleviated.

### 4.3 Integration of Multiple Traits Supports the Utility of the Composite D Value as an Exploratory Low‐P Tolerance Metric

Low‐P tolerance is a complex trait that emerges from the coordinated functioning of multiple morphological, physiological, and rhizosphere processes, rather than from any single dominant trait. Consequently, reliance on individual indicators, such as biomass accumulation, root size, or nutrient‐use efficiency alone, often fails to capture the multifaceted nature of plant adaptation to P deficiency (Heuer et al. 2017). Here, the composite D value integrates growth performance, phosphorus‐use traits, root architectural characteristics, and root functional traits, providing a holistic framework for evaluating genotypic low‐P tolerance. The utility of the D‐value is supported by its associations with key low‐P–responsive traits across both CB and WB. Under P deficiency, D value was positively correlated with PPUE, shoot and root biomass, root system development (TRL and RSA), and rhizosphere functional traits, including citrate, malate, and total carboxylate exudation (Figure 6). The coherence of these correlations integrates the key trait dimensions associated with low‐P performance, rather than being driven by any single component. Importantly, the broadly similar correlation structures observed in CB and WB suggest that the D value reflects common trait associations underlying low‐P adaptation in both domesticated and wild barley. Multivariate approaches that integrate multiple traits have been increasingly recognized as essential for dissecting complex stress tolerances in crops (Alemu et al. 2024). Similar composite indices have successfully been used to rank drought, flooding, and nutrient stress tolerance by combining physiological and morphological traits into a single quantitative metric (Miao et al. 2022; Zhao et al. 2022; Li et al. 2025). Such approaches are particularly valuable for traits like P‐use efficiency, where trade‐offs among acquisition, utilization, and allocation processes often obscure clear relationships when traits are analyzed in isolation (Lambers 2022; Lu et al. 2024). By integrating both aboveground and belowground traits, the D value in this study provides a biologically meaningful and statistically robust tool for ranking low‐P tolerance in barley. It should be noted that because D values were calculated separately within CB and WB groups, direct quantitative comparisons of D values between the CB and WB groups are not appropriate; the index is primarily informative for ranking genotypes within the CB or WB groups. It is also acknowledged that weighting by coefficient of variation reflects the relative variability of each trait across the genotype panel rather than an a priori measure of biological importance; traits showing greater genotypic variation receive higher weights, which may not necessarily correspond to their functional significance in low‐P adaptation. This integrative framework not only facilitates the identification of contrasting genotypes for mechanistic studies but also offers a practical metric for selecting parental lines in breeding programs targeting improved P‐use efficiency under low‐input conditions.

### 4.4 Implication for Barley Breeding for Low‐P Environments

A key insight emerging from this study is that breeding for low‐P tolerance in barley may benefit from moving beyond single‐trait optimization towards simultaneously considering rhizosphere P mobilisation and internal P‐use efficiency across the rhizosphere–plant continuum. Our results show that superior performance under P deficiency is not determined by enhanced root size, total P uptake, or citrate exudation alone, but by the degree to which rhizosphere P mobilization is functionally aligned with internal P‐use efficiency. This suggests that the effectiveness of a trait depends less on its absolute magnitude than on how well it is synchronized with downstream metabolic demand. This insight suggests potential limitations of conventional breeding strategies that target individual belowground traits, such as root length or organic anion release, in isolation. Empirical and modelling studies increasingly indicate that such single‐trait selection can lead to diminishing returns or even maladaptation if the internal nutrient economy is not simultaneously optimized (Lynch 2019). In contrast, plants adapted to nutrient‐poor ecosystems often show complementary functional advantages in acquisition and internal utilization that together maximize growth per unit nutrient invested (Reich 2014). Our findings suggest that wild barley exhibits a similar functional complementarity, with higher PPUE and elevated citrate exudation representing two distinct but functionally aligned mechanisms under P deficiency, rather than a broad reorganization of the whole plant phenotype. From a practical perspective, the composite D value offers a potentially useful framework for multi‐trait evaluation in future breeding. Rather than selecting for extreme phenotypes in individual traits, multivariate indices of this kind could assist breeders in identifying genotypes that achieve an optimal balance among rhizosphere function, internal P economy, and growth. This approach is particularly relevant for low‐input and resource‐constrained agroecosystems, where fertilizer availability is limited, and yield stability depends on efficiency rather than responsiveness. Several important limitations should be acknowledged before these physiological insights could be considered for translational application. The present study was conducted under controlled greenhouse conditions using a soil‐sand mixture with only two P levels and a panel of 20 genotypes, which may not fully capture the complexity of field soil environments, including spatially heterogeneous P distribution, interactions with other nutrients, and diverse microbial communities. Furthermore, the genotype panel, while selected from a previous screening experiment involving 96 genotypes and representing contrasting low‐P performance, may still be limited relative to the full genetic diversity of CB and WB (Tao et al. 2026). Validation of the identified trait relationships across diverse field environments, multiple growing seasons, and a broader germplasm collection will therefore be essential before integrated trait‐based selection can be confidently recommended as a breeding strategy. As global agriculture moves towards reduced P inputs, incorporating integrated trait‐based selection strategies, informed by wild germplasm, could contribute meaningfully to sustaining barley productivity under future nutrient constraints.

## Author Contributions

**Yunpeng Tao:** conceptualization, methodology, investigation, data curation, formal analysis, visualization, writing – original draft. **Xiaowen Fan:** investigation, data curation, formal analysis, visualization, writing – review and editing. **Tahmina Nazish:** investigation, methodology. **Jiayin Pang:** conceptualization, methodology, supervision, resources, writing – review and editing. **Meixue Zhou:** resources, writing – review and editing. **Fanrong Zeng:** resources, writing – review and editing. **Zhong‐Hua Chen:** conceptualization, supervision, writing – review and editing. **Sergey Shabala:** conceptualization, funding acquisition, project administration, supervision, writing – review and editing.

## Funding

This work was supported by Australian Research Council grant LP210200955.

## Supporting information


**Figure S1:** Leaf SPAD value of 10 domesticated barley (CB) accessions and another 10 wild barley (WB) accessions grown under low phosphorus (P5) and moderate phosphorus (P20) conditions for 40 days. Bars represent individual genotype, with dashed horizontal lines indicating the mean value for each species × phosphorus level combination. Statistical significance of the main effects of phosphorus level (P), species (S), and their interaction (*P* × S) was tested using a two‐factorial nested analysis of variance, with genotype nested within species as a random effect. Significance levels are indicated as: ***p < 0.001; *p < 0.05; ns, not significant.
**Figure S2:** Acid phosphatase activity of 10 domesticated barley (CB) accessions and another 10 wild barley (WB) accessions grown under low phosphorus (P5) and moderate phosphorus (P20) conditions for 40 days. Bars represent individual genotype, and dashed horizontal lines indicate the mean value for each species × phosphorus level combination. The significance of the main effects of phosphorus level (P), species (S), and their interaction (*P* × S) was assessed using a two‐factorial nested analysis of variance, with genotype nested within species as a random effect. Significance levels are indicated as: ***p < 0.001; ns, not significant.

## Data Availability

The data that support the findings of this study are available on request from the corresponding author. The data are not publicly available due to privacy or ethical restrictions.
