# Supplementary material for: Wild Barley Exhibits Higher Phosphorus‐Use Efficiency and Greater Rhizosheath Carboxylates Than Cultivated Barley Under Low‐Phosphorus Conditions
Source: Physiol Plant. 2026 Jun 30;178(4):e70990. doi: 10.1111/ppl.70990 (PMC13318851; doi:10.1111/ppl.70990)
Supplement: Supplementary file 1 — Figure S1: Leaf SPAD value of 10 domesticated barley (CB) accessions and another 10 wild barley (WB) accessions grown under low phosphorus (P5) and moderate phosphorus (P20) conditions for 40 days. Bars represent individual genotype, with dashed horizontal lines indicating the mean value for each species × phosphorus level combination. Statistical significance of the main effects of phosphorus level (P), species (S), and their interaction (P × S) was tested using a two‐factorial nested analysis of variance, with genotype nested within species as a random effect. Significance levels are indicated as: ***p < 0.001; *p < 0.05; ns, not significant. Figure S2: Acid phosphatase activity of 10 domesticated barley (CB) accessions and another 10 wild barley (WB) accessions grown under low phosphorus (P5) and moderate phosphorus (P20) conditions for 40 days. Bars represent individual genotype, and dashed horizontal lines indicate the mean value for each species × phosphorus level combination. The significance of the main effects of phosphorus level (P), species (S), and their interaction (P × S) was assessed using a two‐factorial nested analysis of variance, with genotype nested within species as a random effect. Significance levels are indicated as: ***p < 0.001; ns, not significant. [file PPL-178-e70990-s001.pdf]

Supplementary data

Wild barley exhibits higher phosphorus-use efficiency and greater rhizosheath carboxylates than cultivated barley under low-phosphorus conditions

Yunpeng Tao [1,#], Xiaowen Fan [1,#], Tahmina Nazish [1], Jiayin Pang [1,2], Meixue Zhou [3], Fanrong Zeng [4], Zhong-Hua Chen [5], Sergey Shabala [1,6,*]

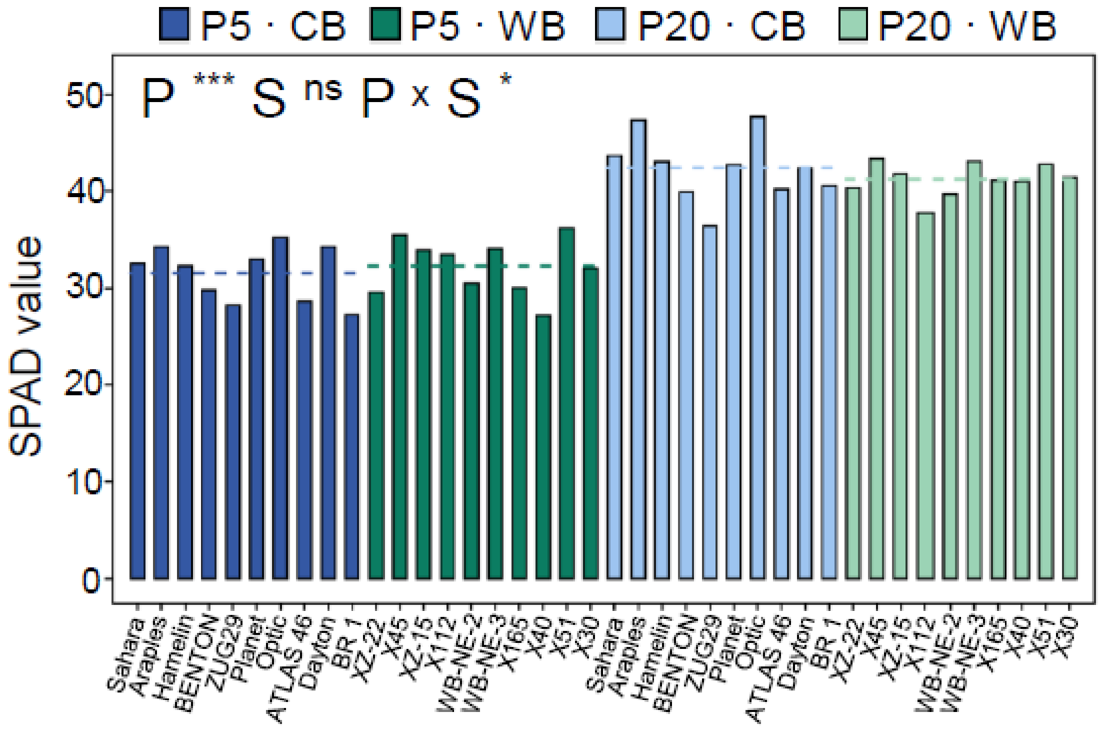

FIGURE S1. Leaf SPAD value of 10 domesticated barley (CB) accessions and another 10 wild barley (WB) accessions grown under low phosphorus (P5) and moderate phosphorus (P20) conditions for 40 days**.** Bars represent individual genotype, with dashed horizontal lines indicating the mean value for each species × phosphorus level combination. Statistical significance of the main effects of phosphorus level (P), species (S), and their interaction (P × S) was tested using a two-factorial nested analysis of variance, with genotype nested within species as a random effect. Significance levels are indicated as: ****P* < 0.001; **P* < 0.05; ns, not significant.

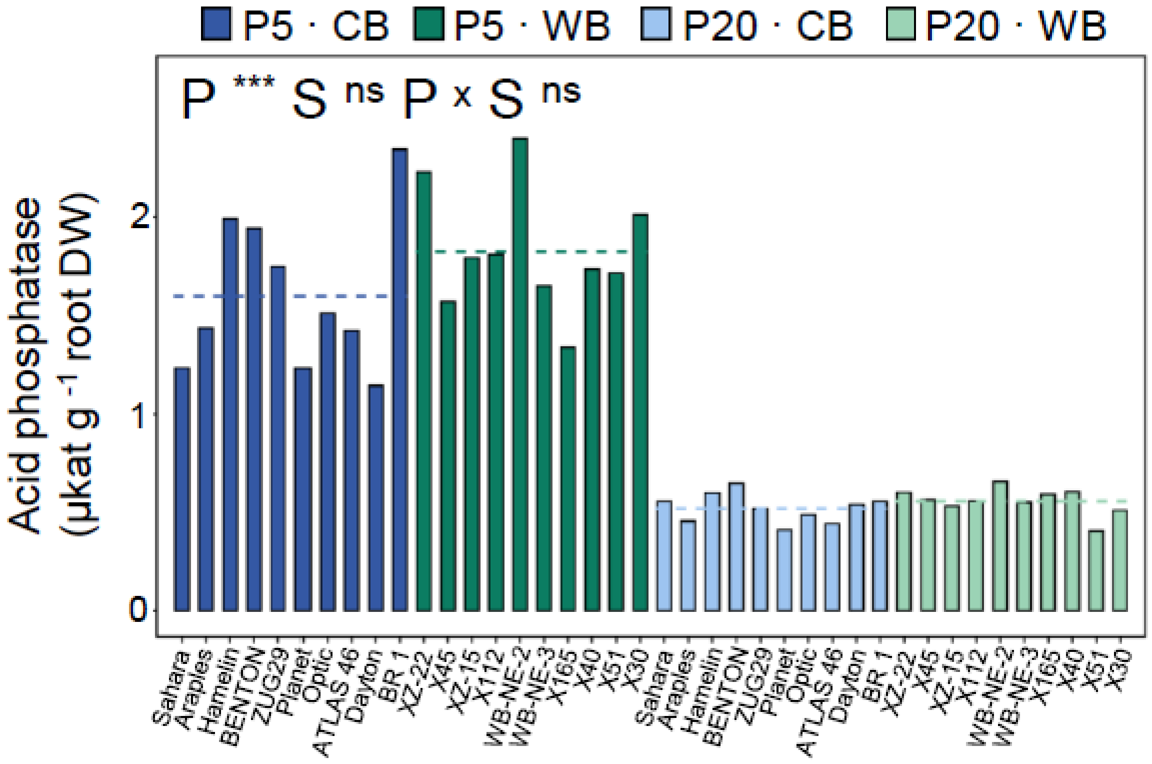

FIGURE S2. Acid phosphatase activity of 10 domesticated barley (CB) accessions and another 10 wild barley (WB) accessions grown under low phosphorus (P5) and moderate phosphorus (P20) conditions for 40 days**.** Bars represent individual genotype, and dashed horizontal lines indicate the mean value for each species × phosphorus level combination. The significance of the main effects of phosphorus level (P), species (S), and their interaction (P × S) was assessed using a two-factorial nested analysis of variance, with genotype nested within species as a random effect. Significance levels are indicated as: ***P < 0.001; ns, not significant.
